# Ethyl 4-(1,3-dioxo-2,3-dihydro-1*H*-benzo[*de*]isoquinolin-2-yl)benzoate

**DOI:** 10.1107/S160053681005049X

**Published:** 2010-12-11

**Authors:** Yi Chen Chan, Abdussalam Salhin, Melati Khairuddean, Madhukar Hemamalini, Hoong-Kun Fun

**Affiliations:** a School of Chemical Sciences, Universiti Sains Malaysia, 11800 USM, Penang, Malaysia; b X-ray Crystallography Unit, School of Physics, Universiti Sains Malaysia, 11800 USM, Penang, Malaysia

## Abstract

The title compound, C_21_H_15_NO_4_, was synthesized by reducing the Schiff base obtained from acenaphthenequinone and ethyl-4-aminobenzoate. The dihedral angle between the essentially planar 1,3-dioxo-2,3-dihydro-1*H*-benzo[*de*]isoquinoline ring system [maximum deviation = 0.061 (2) Å] and the benzene ring is 75.08 (10)°. In the crystal, mol­ecules are connected *via* weak inter­molecular C—H⋯O hydrogen bonds, forming a two-dimensional network. The ethyl group is disordered over two sets of sites with a refined occupancy ratio of 0.502 (12):0.498 (12).

## Related literature

For details and applications of acenaphthenquinone-based Schiff bases, see: Maldanis *et al.* (2002 ▶); Son *et al.* (2006 ▶); Mhaidat *et al.* (2009 ▶); Rodriguez-Argüelles *et al.* (1997 ▶); McDavid *et al.* (1951 ▶); Salhin *et al.* (2007 ▶, 2008 ▶, 2009 ▶); Tameem *et al.* (2006 ▶, 2007 ▶, 2008 ▶); Shalash *et al.* (2010 ▶). For the stability of the temperature controller used in the data collection, see: Cosier & Glazer (1986 ▶).
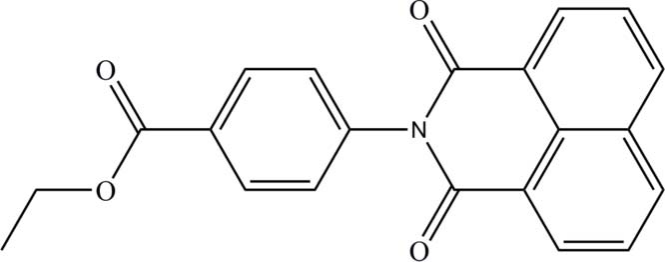


## Experimental

### 

#### Crystal data

C_21_H_15_NO_4_
*M* *_r_* = 345.34
Monoclinic, 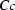

*a* = 5.2025 (7) Å
*b* = 18.066 (3) Å
*c* = 17.560 (2) Å
β = 98.365 (2)°
*V* = 1632.8 (4) Å^3^
*Z* = 4
Mo *K*α radiation
μ = 0.10 mm^−1^
*T* = 100 K
0.49 × 0.21 × 0.08 mm

#### Data collection

Bruker APEXII DUO CCD area-detector diffractometer
Absorption correction: multi-scan (*SADABS*; Bruker, 2009 ▶) *T*_min_ = 0.953, *T*_max_ = 0.992
15726 measured reflections
2393 independent reflections
2157 reflections with *I* > 2σ(*I*)
*R*_int_ = 0.040

#### Refinement

*R*[*F* ^2^ > 2σ(*F* ^2^)] = 0.065
*wR*(*F* ^2^) = 0.171
*S* = 1.07
2393 reflections
256 parameters
4 restraints
H-atom parameters constrained
Δρ_max_ = 0.56 e Å^−3^
Δρ_min_ = −0.38 e Å^−3^

### 

Data collection: *APEX2* (Bruker, 2009 ▶); cell refinement: *SAINT* (Bruker, 2009 ▶); data reduction: *SAINT*; program(s) used to solve structure: *SHELXTL* (Sheldrick, 2008 ▶); program(s) used to refine structure: *SHELXTL*; molecular graphics: *SHELXTL*; software used to prepare material for publication: *SHELXTL* and *PLATON* (Spek, 2009 ▶).

## Supplementary Material

Crystal structure: contains datablocks global, I. DOI: 10.1107/S160053681005049X/lh5180sup1.cif

Structure factors: contains datablocks I. DOI: 10.1107/S160053681005049X/lh5180Isup2.hkl

Additional supplementary materials:  crystallographic information; 3D view; checkCIF report

## Figures and Tables

**Table 1:** Hydrogen-bond geometry (Å, °)

*D*—H⋯*A*	*D*—H	H⋯*A*	*D*⋯*A*	*D*—H⋯*A*
C7—H7*A*⋯O3^i^	0.93	2.60	3.249 (4)	127
C14—H14*A*⋯O1^ii^	0.93	2.41	3.312 (3)	165

Symmetry codes: (i) 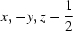; (ii) 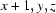.

**Fig. 1.**
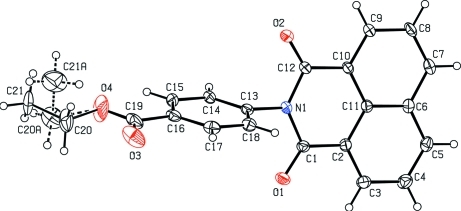
The molecular structure of the title compound, showing 30% probability displacement ellipsoids and the atom-numbering scheme. Dotted lines represents the disorder component.

**Fig. 2.**
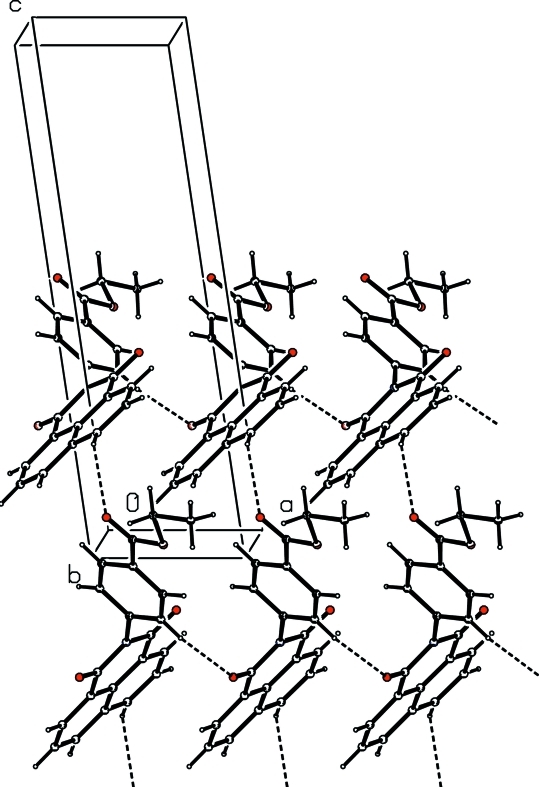
The crystal packing of the title compound with hydrogen bonds shown as dashed lines.

## supplementary crystallographic information

### Comment

Acenaphthenequinone-based Schiff bases have been widely synthesized due to their significant applications  in chemistry (Maldanis *et al.*, 2002; Son *et al.*, 2006), physics (Mhaidat *et al.*, 2009) and pharmacology (Rodriguez-Argüelles *et al.*, 1997; McDavid *et al.*, 1951). As a continuation of the interest of our research group on the synthesis of Schiff base derivatives (Salhin *et al.*, 2007, 2008, 2009; Tameem *et al.*, 2006, 2007, 2008; Shalash *et al.*, 2010), the title compound was prepared through the reduction of the Schiff base which was obtained from the condensation reaction of acenaphthenequinone and ethyl-4-aminobenzoate.

The molecular structure of the title compound is shown in Fig. 1. The 1,3-dioxo-1*H*-benzo[*de*]isoquinoline (O1–O2/N1/C1–C12) ring is approximately planar with maximum deviation of 0.061 (2) Å for atom N1. The ethyl group is disordered over two sites with a refined occupancy ratio of 0.502 (12):0.498 (12).  The dihedral angle between the 1,3-dioxo-1*H*-benzo[de]isoquinoline (O1–O2/N1/C1–C12) ring and the benzene (C13–C18) ring is 75.08 (10)°.

In the crystal structure (Fig. 2), adjacent molecules are connected via intermolecular C7—H7A···O3^i^ and C14—H14A···O1^ii^ (Table 1) hydrogen bonds to form a two-dimensional network.

### Experimental

A mixture of acenaphthenequinone (0.182 g, 1 mmol), ethyl-4-aminobenzoate (0.165 g, 1 mmol) and methanol (30 mL) was allowed to reflux for overnight. The synthesized Schiff base was then reduced using NaBH_4_ in ethanol with stirring at room temperature. Crystal of (I) suitable for X-ray crystallography was obtained by recrystallization from ethanol.

### Refinement

All H atoms were positioned geometrically [C–H = 0.93 or 0.96 Å] and were refined using a riding model, with *U*_iso_(H) = 1.2 or 1.5 *U*_eq_(C). The ethyl group disordered over two sites with a refined occupancy ratio of 0.502 (12):0.498 (12). Since there is no significant anomalous dispersion, 2242 Friedel pairs were merged before the final refinement. The larger than normal displacement parameter of O4 was noticed but it did not improve the precision of the structure to include this atom as a split atom in a disorder model.

### Crystal data

**Table:**

C_21_H_15_NO_4_	*F*(000) = 720
*M**_r_* = 345.34	*D*_x_ = 1.405 Mg m^−^^3^
Monoclinic, *C**c*	Mo *K*α radiation, λ = 0.71073 Å
Hall symbol: C -2yc	Cell parameters from 5950 reflections
*a* = 5.2025 (7) Å	θ = 2.5–30.0°
*b* = 18.066 (3) Å	µ = 0.10 mm^−^^1^
*c* = 17.560 (2) Å	*T* = 100 K
β = 98.365 (2)°	Needle, colourless
*V* = 1632.8 (4) Å^3^	0.49 × 0.21 × 0.08 mm
*Z* = 4	

### Data collection

**Table:**

Bruker APEXII DUO CCD area-detector diffractometer	2393 independent reflections
Radiation source: fine-focus sealed tube	2157 reflections with *I* > 2σ(*I*)
graphite	*R*_int_ = 0.040
φ and ω scans	θ_max_ = 30.0°, θ_min_ = 2.3°
Absorption correction: multi-scan (*SADABS*; Bruker, 2009)	*h* = −7→7
*T*_min_ = 0.953, *T*_max_ = 0.992	*k* = −25→25
15726 measured reflections	*l* = −24→24

### Refinement

**Table:**

Refinement on *F*^2^	Primary atom site location: structure-invariant direct methods
Least-squares matrix: full	Secondary atom site location: difference Fourier map
*R*[*F*^2^ > 2σ(*F*^2^)] = 0.065	Hydrogen site location: inferred from neighbouring sites
*wR*(*F*^2^) = 0.171	H-atom parameters constrained
*S* = 1.07	*w* = 1/[σ^2^(*F*_o_^2^) + (0.0659*P*)^2^ + 3.4903*P*] where *P* = (*F*_o_^2^ + 2*F*_c_^2^)/3
2393 reflections	(Δ/σ)_max_ < 0.001
256 parameters	Δρ_max_ = 0.56 e Å^−^^3^
4 restraints	Δρ_min_ = −0.38 e Å^−^^3^

### Special details

**Table:**

Experimental. The crystal was placed in the cold stream of an Oxford Cryosystems Cobra open-flow nitrogen cryostat (Cosier & Glazer, 1986) operating at 100.0 (1) K.
Geometry. All s.u.'s (except the s.u. in the dihedral angle between two l.s. planes) are estimated using the full covariance matrix. The cell s.u.'s are taken into account individually in the estimation of s.u.'s in distances, angles and torsion angles; correlations between s.u.'s in cell parameters are only used when they are defined by crystal symmetry. An approximate (isotropic) treatment of cell s.u.'s is used for estimating s.u.'s involving l.s. planes.
Refinement. Refinement of F^2^ against ALL reflections. The weighted R-factor wR and goodness of fit S are based on F^2^, conventional R-factors R are based on F, with F set to zero for negative F^2^. The threshold expression of F^2^ > 2σ(F^2^) is used only for calculating R-factors(gt) etc. and is not relevant to the choice of reflections for refinement. R-factors based on F^2^ are statistically about twice as large as those based on F, and R- factors based on ALL data will be even larger.

### Fractional atomic coordinates and isotropic or equivalent isotropic displacement parameters (Å^2^)

**Table:**

	*x*	*y*	*z*	*U*_iso_*/*U*_eq_	Occ. (<1)
O1	0.6572 (6)	0.08698 (16)	0.20848 (18)	0.0348 (6)	
O2	1.3670 (6)	−0.02019 (16)	0.3453 (2)	0.0378 (7)	
O3	0.9558 (11)	0.3058 (3)	0.5083 (2)	0.0746 (15)	
O4	1.2949 (12)	0.3339 (3)	0.4533 (4)	0.105 (2)	
C1	0.7860 (7)	0.0310 (2)	0.2216 (2)	0.0259 (7)	
C2	0.7297 (7)	−0.0388 (2)	0.1797 (2)	0.0271 (7)	
C3	0.5174 (8)	−0.0424 (2)	0.1229 (2)	0.0336 (8)	
H3A	0.4150	−0.0007	0.1106	0.040*	
C4	0.4555 (9)	−0.1099 (3)	0.0835 (3)	0.0386 (9)	
H4A	0.3117	−0.1125	0.0453	0.046*	
C5	0.6070 (8)	−0.1716 (2)	0.1013 (2)	0.0334 (8)	
H5A	0.5639	−0.2158	0.0753	0.040*	
C6	0.8256 (7)	−0.1689 (2)	0.1583 (2)	0.0293 (8)	
C7	0.9882 (8)	−0.2308 (2)	0.1776 (2)	0.0332 (8)	
H7A	0.9477	−0.2756	0.1527	0.040*	
C8	1.2041 (9)	−0.2262 (2)	0.2323 (3)	0.0338 (8)	
H8A	1.3093	−0.2674	0.2440	0.041*	
C9	1.2662 (8)	−0.1586 (2)	0.2707 (2)	0.0293 (7)	
H9A	1.4141	−0.1554	0.3073	0.035*	
C10	1.1111 (7)	−0.09719 (19)	0.2547 (2)	0.0254 (7)	
C11	0.8898 (7)	−0.1011 (2)	0.1980 (2)	0.0245 (7)	
C12	1.1773 (7)	−0.0277 (2)	0.2972 (2)	0.0267 (7)	
N1	1.0028 (6)	0.03130 (17)	0.27965 (19)	0.0254 (6)	
C13	1.0402 (7)	0.0969 (2)	0.3273 (2)	0.0262 (7)	
C14	1.2342 (7)	0.1468 (2)	0.3184 (2)	0.0290 (7)	
H14A	1.3454	0.1382	0.2824	0.035*	
C15	1.2614 (8)	0.2100 (2)	0.3639 (3)	0.0345 (9)	
H15A	1.3917	0.2440	0.3587	0.041*	
C16	1.0928 (9)	0.2223 (2)	0.4175 (2)	0.0358 (9)	
C17	0.9001 (8)	0.1711 (3)	0.4258 (2)	0.0363 (9)	
H17A	0.7891	0.1793	0.4619	0.044*	
C18	0.8716 (8)	0.1078 (2)	0.3807 (2)	0.0322 (8)	
H18A	0.7424	0.0735	0.3861	0.039*	
C19	1.1063 (12)	0.2905 (3)	0.4660 (3)	0.0525 (14)	
C20	1.244 (3)	0.4000 (7)	0.5078 (9)	0.065 (4)	0.493 (17)
H20A	1.0810	0.4252	0.4906	0.078*	0.493 (17)
H20B	1.2509	0.3847	0.5610	0.078*	0.493 (17)
C21	1.483 (3)	0.4465 (6)	0.4943 (9)	0.066 (4)	0.493 (17)
H21A	1.4944	0.4896	0.5267	0.098*	0.493 (17)
H21B	1.4645	0.4615	0.4414	0.098*	0.493 (17)
H21C	1.6374	0.4173	0.5066	0.098*	0.493 (17)
C20A	1.343 (4)	0.4184 (10)	0.4745 (8)	0.074 (5)	0.507 (17)
H20C	1.4622	0.4431	0.4452	0.088*	0.507 (17)
H20D	1.1854	0.4470	0.4749	0.088*	0.507 (17)
C21A	1.470 (3)	0.3911 (11)	0.5553 (10)	0.103 (7)	0.507 (17)
H21D	1.4901	0.4322	0.5904	0.155*	0.507 (17)
H21E	1.6372	0.3701	0.5517	0.155*	0.507 (17)
H21F	1.3610	0.3543	0.5737	0.155*	0.507 (17)

### Atomic displacement parameters (Å^2^)

**Table:**

	*U*^11^	*U*^22^	*U*^33^	*U*^12^	*U*^13^	*U*^23^
O1	0.0360 (15)	0.0291 (14)	0.0376 (15)	0.0107 (12)	−0.0006 (12)	−0.0021 (11)
O2	0.0347 (15)	0.0267 (14)	0.0483 (17)	0.0047 (11)	−0.0064 (13)	−0.0026 (12)
O3	0.126 (4)	0.056 (2)	0.043 (2)	0.035 (3)	0.014 (2)	−0.0126 (18)
O4	0.102 (4)	0.056 (3)	0.152 (6)	−0.014 (3)	0.003 (4)	−0.071 (3)
C1	0.0237 (15)	0.0251 (17)	0.0286 (17)	0.0035 (12)	0.0027 (13)	−0.0016 (13)
C2	0.0257 (16)	0.0246 (17)	0.0311 (18)	−0.0002 (13)	0.0050 (14)	−0.0010 (13)
C3	0.0293 (19)	0.034 (2)	0.036 (2)	0.0037 (15)	−0.0017 (16)	−0.0033 (16)
C4	0.034 (2)	0.042 (2)	0.037 (2)	0.0004 (17)	−0.0010 (17)	−0.0098 (18)
C5	0.0343 (19)	0.0318 (19)	0.0340 (19)	−0.0042 (16)	0.0048 (16)	−0.0082 (15)
C6	0.0308 (18)	0.0262 (18)	0.0315 (18)	−0.0034 (14)	0.0069 (15)	−0.0025 (14)
C7	0.042 (2)	0.0202 (16)	0.039 (2)	−0.0004 (15)	0.0101 (17)	−0.0030 (14)
C8	0.041 (2)	0.0174 (16)	0.043 (2)	0.0069 (15)	0.0071 (17)	−0.0006 (15)
C9	0.0309 (18)	0.0197 (15)	0.0367 (19)	0.0027 (13)	0.0023 (15)	0.0004 (14)
C10	0.0268 (17)	0.0191 (15)	0.0306 (17)	0.0019 (12)	0.0053 (14)	0.0026 (13)
C11	0.0241 (16)	0.0217 (15)	0.0283 (16)	−0.0006 (12)	0.0054 (13)	−0.0006 (12)
C12	0.0269 (16)	0.0202 (15)	0.0324 (17)	0.0021 (13)	0.0024 (13)	0.0033 (13)
N1	0.0261 (14)	0.0195 (13)	0.0302 (15)	0.0014 (11)	0.0028 (12)	−0.0017 (11)
C13	0.0255 (16)	0.0226 (16)	0.0295 (17)	0.0059 (13)	0.0008 (13)	−0.0019 (13)
C14	0.0282 (17)	0.0219 (16)	0.0370 (19)	0.0033 (13)	0.0052 (14)	−0.0028 (14)
C15	0.0288 (18)	0.0224 (17)	0.051 (2)	0.0011 (14)	0.0009 (17)	−0.0081 (16)
C16	0.041 (2)	0.0303 (19)	0.0328 (19)	0.0131 (16)	−0.0068 (16)	−0.0080 (15)
C17	0.039 (2)	0.041 (2)	0.0277 (18)	0.0122 (17)	0.0023 (16)	−0.0033 (16)
C18	0.0335 (19)	0.033 (2)	0.0305 (18)	0.0038 (15)	0.0059 (15)	0.0003 (15)
C19	0.075 (4)	0.032 (2)	0.044 (2)	0.021 (2)	−0.013 (2)	−0.0114 (19)
C20	0.092 (11)	0.041 (6)	0.058 (8)	−0.012 (6)	0.003 (7)	−0.022 (6)
C21	0.079 (9)	0.033 (5)	0.085 (10)	−0.031 (6)	0.014 (8)	−0.014 (5)
C20A	0.082 (12)	0.074 (11)	0.068 (9)	0.014 (9)	0.023 (8)	−0.015 (8)
C21A	0.068 (11)	0.102 (14)	0.15 (2)	0.012 (9)	0.047 (12)	0.023 (13)

### Geometric parameters (Å, °)

**Table:**

O1—C1	1.217 (5)	C12—N1	1.405 (4)
O2—C12	1.210 (5)	N1—C13	1.448 (5)
O3—C19	1.188 (7)	C13—C14	1.379 (5)
O4—C19	1.300 (9)	C13—C18	1.387 (5)
O4—C20	1.576 (14)	C14—C15	1.389 (5)
O4—C20A	1.581 (19)	C14—H14A	0.9300
C1—N1	1.406 (5)	C15—C16	1.394 (6)
C1—C2	1.468 (5)	C15—H15A	0.9300
C2—C3	1.377 (5)	C16—C17	1.387 (7)
C2—C11	1.410 (5)	C16—C19	1.494 (6)
C3—C4	1.417 (6)	C17—C18	1.386 (6)
C3—H3A	0.9300	C17—H17A	0.9300
C4—C5	1.375 (6)	C18—H18A	0.9300
C4—H4A	0.9300	C20—C21	1.546 (10)
C5—C6	1.402 (6)	C20—H20A	0.9700
C5—H5A	0.9300	C20—H20B	0.9700
C6—C7	1.415 (6)	C21—H21A	0.9600
C6—C11	1.424 (5)	C21—H21B	0.9600
C7—C8	1.370 (6)	C21—H21C	0.9600
C7—H7A	0.9300	C20A—C21A	1.555 (10)
C8—C9	1.409 (5)	C20A—H20C	0.9700
C8—H8A	0.9300	C20A—H20D	0.9700
C9—C10	1.376 (5)	C21A—H21D	0.9600
C9—H9A	0.9300	C21A—H21E	0.9600
C10—C11	1.410 (5)	C21A—H21F	0.9600
C10—C12	1.476 (5)		
			
C19—O4—C20	98.9 (7)	C1—N1—C13	116.7 (3)
C19—O4—C20A	129.7 (8)	C14—C13—C18	122.0 (4)
C20—O4—C20A	33.0 (6)	C14—C13—N1	120.5 (3)
O1—C1—N1	119.7 (3)	C18—C13—N1	117.5 (3)
O1—C1—C2	123.7 (3)	C13—C14—C15	119.1 (4)
N1—C1—C2	116.6 (3)	C13—C14—H14A	120.5
C3—C2—C11	120.8 (3)	C15—C14—H14A	120.5
C3—C2—C1	118.9 (3)	C14—C15—C16	119.9 (4)
C11—C2—C1	120.2 (3)	C14—C15—H15A	120.1
C2—C3—C4	119.7 (4)	C16—C15—H15A	120.1
C2—C3—H3A	120.1	C17—C16—C15	120.0 (4)
C4—C3—H3A	120.1	C17—C16—C19	117.7 (4)
C5—C4—C3	120.3 (4)	C15—C16—C19	122.3 (5)
C5—C4—H4A	119.8	C18—C17—C16	120.6 (4)
C3—C4—H4A	119.8	C18—C17—H17A	119.7
C4—C5—C6	120.8 (4)	C16—C17—H17A	119.7
C4—C5—H5A	119.6	C17—C18—C13	118.5 (4)
C6—C5—H5A	119.6	C17—C18—H18A	120.8
C5—C6—C7	122.5 (4)	C13—C18—H18A	120.8
C5—C6—C11	119.3 (3)	O3—C19—O4	123.3 (5)
C7—C6—C11	118.2 (3)	O3—C19—C16	124.6 (6)
C8—C7—C6	121.4 (4)	O4—C19—C16	112.0 (5)
C8—C7—H7A	119.3	C21—C20—O4	96.2 (9)
C6—C7—H7A	119.3	C21—C20—H20A	112.5
C7—C8—C9	119.7 (4)	O4—C20—H20A	112.5
C7—C8—H8A	120.1	C21—C20—H20B	112.5
C9—C8—H8A	120.1	O4—C20—H20B	112.5
C10—C9—C8	121.0 (4)	H20A—C20—H20B	110.0
C10—C9—H9A	119.5	C21A—C20A—O4	86.6 (13)
C8—C9—H9A	119.5	C21A—C20A—H20C	114.2
C9—C10—C11	119.8 (3)	O4—C20A—H20C	114.2
C9—C10—C12	119.7 (3)	C21A—C20A—H20D	114.2
C11—C10—C12	120.4 (3)	O4—C20A—H20D	114.2
C2—C11—C10	121.0 (3)	H20C—C20A—H20D	111.4
C2—C11—C6	119.1 (3)	C20A—C21A—H21D	109.5
C10—C11—C6	119.9 (3)	C20A—C21A—H21E	109.5
O2—C12—N1	120.3 (3)	H21D—C21A—H21E	109.5
O2—C12—C10	123.7 (3)	C20A—C21A—H21F	109.5
N1—C12—C10	116.0 (3)	H21D—C21A—H21F	109.5
C12—N1—C1	125.5 (3)	H21E—C21A—H21F	109.5
C12—N1—C13	117.8 (3)		
			
O1—C1—C2—C3	−1.1 (6)	C10—C12—N1—C1	−5.3 (5)
N1—C1—C2—C3	179.1 (4)	O2—C12—N1—C13	−7.6 (5)
O1—C1—C2—C11	179.5 (4)	C10—C12—N1—C13	171.5 (3)
N1—C1—C2—C11	−0.3 (5)	O1—C1—N1—C12	−175.6 (4)
C11—C2—C3—C4	1.3 (6)	C2—C1—N1—C12	4.3 (5)
C1—C2—C3—C4	−178.1 (4)	O1—C1—N1—C13	7.6 (5)
C2—C3—C4—C5	−0.3 (7)	C2—C1—N1—C13	−172.6 (3)
C3—C4—C5—C6	−0.4 (7)	C12—N1—C13—C14	76.6 (4)
C4—C5—C6—C7	−179.1 (4)	C1—N1—C13—C14	−106.3 (4)
C4—C5—C6—C11	0.1 (6)	C12—N1—C13—C18	−104.4 (4)
C5—C6—C7—C8	178.5 (4)	C1—N1—C13—C18	72.7 (4)
C11—C6—C7—C8	−0.8 (6)	C18—C13—C14—C15	−0.3 (6)
C6—C7—C8—C9	0.4 (6)	N1—C13—C14—C15	178.6 (3)
C7—C8—C9—C10	0.8 (6)	C13—C14—C15—C16	−0.2 (6)
C8—C9—C10—C11	−1.4 (6)	C14—C15—C16—C17	0.7 (6)
C8—C9—C10—C12	178.5 (4)	C14—C15—C16—C19	−177.7 (4)
C3—C2—C11—C10	178.4 (4)	C15—C16—C17—C18	−0.6 (6)
C1—C2—C11—C10	−2.2 (5)	C19—C16—C17—C18	177.9 (4)
C3—C2—C11—C6	−1.6 (5)	C16—C17—C18—C13	0.1 (6)
C1—C2—C11—C6	177.8 (3)	C14—C13—C18—C17	0.3 (6)
C9—C10—C11—C2	−179.0 (4)	N1—C13—C18—C17	−178.6 (3)
C12—C10—C11—C2	1.1 (5)	C20—O4—C19—O3	0.3 (10)
C9—C10—C11—C6	1.0 (5)	C20A—O4—C19—O3	−13.0 (13)
C12—C10—C11—C6	−179.0 (3)	C20—O4—C19—C16	176.7 (7)
C5—C6—C11—C2	0.8 (5)	C20A—O4—C19—C16	163.4 (9)
C7—C6—C11—C2	−179.9 (4)	C17—C16—C19—O3	−4.0 (7)
C5—C6—C11—C10	−179.1 (4)	C15—C16—C19—O3	174.4 (5)
C7—C6—C11—C10	0.2 (5)	C17—C16—C19—O4	179.7 (5)
C9—C10—C12—O2	1.7 (6)	C15—C16—C19—O4	−1.9 (7)
C11—C10—C12—O2	−178.4 (4)	C19—O4—C20—C21	176.4 (10)
C9—C10—C12—N1	−177.4 (3)	C20A—O4—C20—C21	−22.5 (12)
C11—C10—C12—N1	2.5 (5)	C19—O4—C20A—C21A	84.2 (13)
O2—C12—N1—C1	175.5 (4)	C20—O4—C20A—C21A	59.5 (14)

### Hydrogen-bond geometry (Å, °)

**Table:**

*D*—H···*A*	*D*—H	H···*A*	*D*···*A*	*D*—H···*A*
C7—H7A···O3^i^	0.93	2.60	3.249 (4)	127
C14—H14A···O1^ii^	0.93	2.41	3.312 (3)	165

Symmetry codes: (i) *x*, −*y*, *z*−1/2; (ii) *x*+1, *y*, *z*.
